# Breaking down KRAS: small-molecule degraders for cancer therapy

**DOI:** 10.1038/s41392-025-02172-4

**Published:** 2025-03-14

**Authors:** Tina Kos, Dieter Saur

**Affiliations:** 1 Chair of Translational Cancer Research and Institute of Experimental Cancer Therapy, Klinikum rechts der Isar, School of Medicine, Technische Universität München, Ismaninger Str. 22, 81675 Munich, Germany; 2 Division of Translational Cancer Research, German Cancer Research Center (DKFZ) and German Cancer Consortium (DKTK), Im Neuenheim Feld 280, 69120 Heidelberg, Germany; 3 Center for Translational Cancer Research (TranslaTUM), School of Medicine, Technical University of Munich, Einsteinstr. 25, 81675 Munich, Germany; 4 Department of Internal Medicine II, Klinikum rechts der Isar, Technische Universität München, Ismaninger Str. 22, 81675 Munich, Germany

**Keywords:** Drug development, Oncogenes

In their recently published study in *Science*, Popow and colleagues developed a proteolysis-targeting chimera (PROTAC) for the in vivo degradation of several oncogenic KRAS variants.^1^ Leveraging detailed biophysical analyses and crystal structures of ternary complexes of candidate ligands for KRAS and the von Hippel-Lindau (VHL) E3 ubiquitin ligase complex, they designed a small molecule capable of potently and selectively targeting 13 of the 17 most common KRAS mutants.

KRAS mutants are among the most prominent oncogenic drivers, and are present in a large proportion of pancreatic, colorectal, and lung cancers. Due to its protein structure, KRAS has been considered undruggable until the discovery of a pocket beneath Switch II,^2^ which spurred the development of novel small molecule KRAS inhibitors (KRASi), most of which are mutation-specific. Two main targeting approaches have emerged: Off-state inhibitors, such as the clinically approved KRAS^G12C^i adagrasib and sotorasib, and the investigational KRAS^G12D^i MRTX1133 that block KRAS in its inactive, GDP-bound state; and On-state inhibitors including the KRAS^G12C^i BBO-8520 and the pan-RASi RMC-6236 that target the GTP-bound state, preventing downstream effector binding and signaling. Permanent covalent inhibitors, such as adagrasib and sotorasib, and the KRAS^G12D^ (On) inhibitor RMC-9805, differ from noncovalent inhibitors like MRTX1133 in their ability to form strong, enduring modifications of their targets. However, the clinically approved inhibitors thus far have shown heterogenous patient responses and emergence of drug resistance. Analysis of relapsed patients revealed a complex landscape of resistance, including genomic *KRAS* amplification, KRAS mutations that reduce drug binding or stabilize the active (On) state, as well as mutations and/or amplifications in upstream or downstream components of the RAS pathway that amplify RAS signaling.^3^

**Fig. 1:**
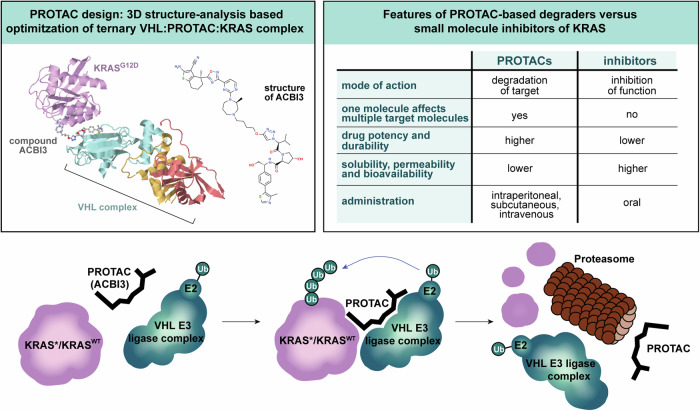
PROTAC-mediated ternary complex formation of the VHL E3 ligase and KRAS enables E2 ubiquitin-conjugating enzyme (E2) induced ubiquitination (Ub) and subsequent proteasomal degradation of mutated (KRAS*) and wild-type KRAS (KRAS^WT^) proteins. The PROTAC linker and the VHL E3 ligase complex are subsequently released and can target additional KRAS molecules. Popow et al. used structural analysis of the ternary complex to optimize the PROTAC linker compound and achieve KRAS degradation and tumor regression in vivo with the final optimized compound ACBI3 (upper left panel; 3D complex and compound structure were extracted from Protein Data Bank ID 8QVU; https://www.ebi.ac.uk/pdbe/entry/pdb/8qvu). PROTACs differ in several key aspects compared to small molecule inhibitors as depicted in the upper right box

To develop a KRAS PROTAC capable of degrading the most common oncogenic KRAS mutants, the authors designed a heterobifunctional small molecule that links KRAS mutant proteins and the VHL E3 ubiquitin ligase complex, prompting ubiquitination and proteasomal degradation of KRAS (Fig. 1). The design was based on the recently described high-affinity KRAS Switch II pocket ligand, BI-2865, which blocked multiple KRAS mutations as a non-covalent pan-KRAS off-state inhibitor.^4^ They utilized the Switch II pocket-binding motif of BI-2865 and x-ray crystallographic structures to identify promising positions for PROTAC linkers that are coupled to the binding motif for the VHL E3 ligase. A screen with alkyl and polyethylene glycol (PEG)–based linkers identified a compound with high cooperativity, which facilitated high-affinity ternary complex formation of VHL:PROTAC:KRAS^G12D^. Testing KRAS degradation across cell lines with different KRAS mutations, 13 out of the 17 most common KRAS mutants as well as wild type (WT) KRAS, were efficiently degraded.

A comparison of the KRAS degrading PROTAC with small molecule KRASi showed that both approaches effectively inhibited KRAS downstream signaling and hindered cell proliferation in KRAS-dependent cell lines, with PROTACs displaying higher potency. Furthermore, cells treated with the PROTAC exhibited prolonged MAPK pathway suppression following treatment cessation, in contrast to KRASi, owing to the degradation of existing KRAS protein. To assess the usability of the degrader in different cancer entities, it was tested against a cohort of 300 cancer cell lines that covered a variety of *KRAS* mutations. This revealed a large overlap in sensitivity between the KRAS degrader and the pan-KRAS small molecule inhibitor BI-2493, albeit with higher potency for the degrader. Cell lines harboring GTP-hydrolysis-impaired mutant KRAS^Q61K^ were insensitive. Phosphoproteomic analysis of two cell lines treated with the degrader and a similar, non-degrading inhibitor revealed largely overlapping phosphorylation profiles, with the degrader demonstrating superior effect size and potency. This advantage over traditional inhibitors highlights the potential of ACBI3 as a promising compound for clinical applications.

In vivo pharmacokinetic profiling of the degrader revealed insufficient plasma concentrations and a reduction in potency in the presence of 10% mouse serum. These findings prompted further optimization, leading to the design of ACBI3 (Fig. 1), which displayed substantially enhanced in vivo activity. Subcutaneous or intraperitoneal injection of ACBI3 blocked tumor growth in immunodeficient mice subcutaneously xenografted with human *KRAS*^G12D^-mutant colorectal carcinoma (GP2d) or *KRAS*^G12V^-mutant ovarian leiomyosarcoma (RKN) cell lines over a 14-day treatment period. Interestingly, a comparison of ACBI3 with the pan-KRAS inhibitor BI-2493 in the RKN model revealed comparable therapeutic efficacy, indicating that—over the 14-days treatment period—KRAS degradation does not confer a significant increase in therapeutic potency over KRASi. However, development of resistance was not investigated, which will be important for clinical translation.

Compared to classical small drug KRASi, which are all expected to be susceptible to primary or acquired resistance, such as increased *KRAS* expression, e.g., via *KRAS* amplification, or secondary *KRAS* mutations,^3^ the PROTAC approach has a fundamental distinct mechanism of action, which is potentially capable of overcoming or at least mitigating some of these resistance mechanisms (Fig. 1). A single PROTAC can irreversibly degrade multiple target molecules, enabling more efficient pathway inactivation compared to inhibitors. However, its dual-binding mechanism introduces several challenges. For example, loss of VHL E3 expression can emerge as a potential additional resistance mechanism. Additionally, the dual-binding design increases the molecular weight and structural complexity of PROTACs, which hinders their solubility, permeability and bioavailability.^5^ Consequently, achieving effective intracellular concentrations requires higher compound doses. Furthermore, unlike the orally available KRAS inhibitors, ACBI3 requires subcutaneous or intraperitoneal injection, presenting a significant challenge for daily, long-term patient use. Efforts to overcome these limitations and improve PROTAC pharmacokinetics are ongoing. Strategies include reducing molecular weight, improving solubility, employing alternative E3 ligases for target degradation, and developing alternative delivery strategies including nanoparticles, liposomes, and antibody- or aptamer-PROTAC conjugates.^5^ Despite the current limitations, several PROTACS have advanced to Phase 3 studies since the first compounds entered clinical trials in 2019, demonstrating their potential as transformative therapeutic agents.^5^ ACBI3 has not yet been evaluated in patients, and further in vitro and in vivo studies are required to advance this new compound to phase 1/2 clinical trials. This includes extended in vivo efficacy and resistance testing beyond the reported 14-day treatment period, pharmacokinetics/pharmacodynamics (PK/PD) and toxicology assessments, as well as Good Manufacturing Practice (GMP) production and an investigational new drug (IND) application.

Once considered undruggable, remarkable progress in targeting oncogenic KRAS has led to more than 30 compounds now in clinical evaluation or approved for treatment of KRAS-mutant cancers. Most inhibitors are mutation-specific and theoretically less toxic than pan-KRAS inhibitors, as they do not inhibit KRAS^WT^ or its homologues NRAS and HRAS. Notably, the pan-RAS (on) inhibitor RMC-6236 showed promising activity and manageable toxicity in a phase 2 clinical trial of advanced second-line metastatic PDAC patients (https://ir.revmed.com/static-files/eeeb0690-0ef4-44b8-b5fe-8d11d8df3c9a). The development of ACBI3 significantly expands the repertoire of direct pan-KRAS inhibitors enabling innovative combinations with mutation-specific approaches to potentially prevent emergence of resistance. While it remains uncertain which strategy will prove most effective, tailored approaches - incorporating immunotherapy and other combinations based on cancer type, tissue origin, KRAS mutations, and downstream effectors – are likely required to achieve durable responses. Ongoing efforts to elucidate and overcome the various mechanisms of primary and acquired resistance to KRAS inhibitors offer a promising path forward for combating KRAS-driven cancers.
